# How and to what end may consciousness contribute to action? Attributing properties of consciousness to an embodied, minimally cognitive artificial neural network

**DOI:** 10.3389/fpsyg.2013.00324

**Published:** 2013-06-18

**Authors:** Holk Cruse, Malte Schilling

**Affiliations:** Center of Excellence ‘Cognitive Interaction Technology’, University of BielefeldBielefeld, Germany

**Keywords:** recurrent neural network, consciousness, minimal cognitive system, motor control, robotic architecture, embodiment, access consciousness, internal body model

## Abstract

An artificial neural network called reaCog is described which is based on a decentralized, reactive and embodied architecture developed to control non-trivial hexapod walking in an unpredictable environment (Walknet) while using insect-like navigation (Navinet). In reaCog, these basic networks are extended in such a way that the complete system, reaCog, adopts the capability of inventing new behaviors and – via internal simulation – of planning ahead. This cognitive expansion enables the reactive system to be enriched with additional procedures. Here, we focus on the question to what extent properties of phenomena to be characterized on a different level of description as for example consciousness can be found in this minimally cognitive system. Adopting a monist view, we argue that the phenomenal aspect of mental phenomena can be neglected when discussing the function of such a system. Under this condition, reaCog is discussed to be equipped with properties as are bottom-up and top-down attention, intentions, volition, and some aspects of Access Consciousness. These properties have not been explicitly implemented but emerge from the cooperation between the elements of the network. The aspects of Access Consciousness found in reaCog concern the above mentioned ability to plan ahead and to invent and guide (new) actions. Furthermore, global accessibility of memory elements, another aspect characterizing Access Consciousness is realized by this network. reaCog allows for both reactive/automatic control and (access-) conscious control of behavior. We discuss examples for interactions between both the reactive domain and the conscious domain. Metacognition or Reflexive Consciousness is not a property of reaCog. Possible expansions are discussed to allow for further properties of Access Consciousness, verbal report on internal states, and for Metacognition. In summary, we argue that already simple networks allow for properties of consciousness if leaving the phenomenal aspect aside.

## Introduction

The nature of the mental, in particular of consciousness, and its relation to the physical world is a fundamental concern in philosophy of mind. Studies addressing this question have led to a variety of views concerning this matter. Vision (2011) reviews a huge number of variations and sub-variations of these views forming a “crowded and messy field” (Vision, 2011, p. 29). Although, as seen by somebody not being an expert in philosophy of mind, most of these views appear to show a large amount of plausibility, the various positions defended by their proponents appear to be characterized by fundamental disagreements, and a commonly agreed solution seems not to be in reach.

Therefore, as a complement to these top-down approaches, in what follows we would like to begin with a quite different approach, a bottom-up approach. The goal of this approach is to develop a neural architecture that shows a number of abilities found in autonomous agents, i.e., the goal is to formulate quantitative hypotheses concerning the structure and functioning of autonomous and perhaps cognitive systems that can be tested on a robot. In this article, such a system will be presented and used as a scaffold for discussions concerning the higher-level properties usually connoted with mental aspects. In particular we can ask to what extent properties may be observed that have not explicitly been implemented and therefore may loosely be termed emergent properties. Specifically, in this context properties are considered that may be related to high-level properties as are attention, intention, volition, or consciousness.

Our goal is not to construct an artificial system that is equipped with, for example, consciousness. Instead, we want to use this system as a tool to test to what extent descriptions of mental phenomena used in psychology or philosophy of mind may be applied to such an artificial system. All these definitions necessarily rely on verbal formulations and are therefore open to different interpretations. In contrast, a definition based on a mathematical formulation or being given in the form of a quantitative simulation does not suffer from such ambiguities. Based on such an explicit definition, the properties of the phenomenon can be studied in detail and judgments are possible whether the specific definition chosen appears to be sufficient or whether critical aspects of the phenomenon of interest are missing. In the latter case, the definition may be improved accordingly. To start with such an approach, we refer to definitions of attention from Desimone and Duncan (1995), of intention from Pacherie (2006) and Goschke (2013), and for volition from Goschke (2013). Concerning consciousness, as discussed by Cleeremans (2005), this phenomenon may only be approachable if the task is split into different aspects that are treated separately. Following Block (1995, 2001), to this end Cleeremans (2005) distinguishes between Access Consciousness, Metacognition, and Phenomenal Consciousness.

To proceed in this way, in Section “reaCog, An Embodied, Minimal Version of a Cognitive System” we will briefly and, as far as required for a basic understanding, explain the essential properties of a system called reaCog that is supposed to be equipped with cognitive abilities while being strongly based on a reactive architecture (Schilling and Cruse, 2008, submitted).

Applying a bottom-up approach we focus on a reactive system that is able to deal with a specific domain of behavior, namely walking with six legs in an unpredictable environment including climbing over very large gaps. The reactive part of the system has been termed “Walknet” and is biologically inspired by detailed work on the walking of the stick insect (Dürr et al., 2004; Bläsing, 2006; Schilling et al., submitted a). The stepping patterns (“gaits”) observed (in the robot as in the insects) are not explicitly implemented but result from the cooperation of local rules and the coupling through the environment. Furthermore, the system has been expanded by a network allowing for insect-like navigation (“Navinet,” Cruse and Wehner, 2011; Hoinville et al., 2012), where the agent is able to select visiting one of a number of food sources learned, and to decide between traveling to the food source or back home. Of particular interest is here that Navinet (like a desert ant) attends known visual landmarks only in the appropriate context, i.e., depending on the food source it is actually traveling to. Furthermore, the reactive network Navinet does not require an explicit “cognitive map” to describe experimental results, for which earlier authors have assumed such a map to be necessary.

The complete network is based on a decentralized architecture consisting of procedural, or reactive, elements which, in turn, consist of artificial neurons. The reactive network, showing a heterarchical structure, allows for selection of different behaviors, which includes protection against, in the actual context, non-relevant sensory input.

As a next “evolutionary” step, the network is equipped with a flexible internal body model allowing for internal simulation of behaviors. This extended system is called reaCog, consisting of the reactive “Walknet” which has been expanded to include cognitive properties. Together with the introduction of this “cognitive expansion,” reaCog comprises the ability to plan ahead and to invent new behaviors in order to solve problems for which no solution is actually available. As such, this cognitive expansion cannot function by itself, but only, like a parasite, operates on top of the reactive structures (Norman and Shallice, 1986). The final decision to store a new behavioral procedure is not purely stochastic, because the proposals made by the cognitive expansion are tested for feasibility via the internal simulation as well as by performing the behavior in reality. Thus, invention of new behaviors may be viewed as to be based on a Darwinian procedure (See General Characteristics of reaCog and A Possible Expansion).

Following the definition of McFarland and Bösser (1993) a cognitive system is characterized by the capability of planning ahead. In this sense, reaCog can be termed a cognitive system, that allows planning ahead via internal simulation. As the cognitive system is crucially dependent on its reactive foundations (therefore the name reaCog), the development of rich cognitive abilities requires a correspondingly rich behavioral repertoire.

After having introduced reaCog in Section “reaCog, An Embodied, Minimal Version of a Cognitive System,” we will, in Section “Properties of reaCog Being Characterized by Applying Other Levels of Description,” discuss to what extent this network, forming a simple structure, could serve as a scaffold providing a quantitative foundation for more abstract concepts formulated on levels of description as being applied in psychology or philosophy of mind. Specifically, we will address the phenomenon of consciousness which, according to some authors, may be an inherent property for at least some cognitive systems. Therefore, although we do not want to state that consciousness should be attributed to our system in any sense, we want to discuss in Section “Properties of reaCog Being Characterized by Applying Other Levels of Description” how properties characterized on different levels of description can be observed in our model. In Section “Phenomenality” we discuss as to how phenomenal aspects might be attributed to physical systems and conclude by arguing that the phenomenal aspect is not crucial for understanding the function. We are not trying to solve the “hard” problem (Chalmers, 1996), but will argue that it suffices to concentrate on the functional aspect.

In Section “Attention, Volition, Intention” we will briefly address the question if terms as attention, intention, or volition might be attributed to our network. In Sections “Access Consciousness” and *“Metacognition”* we will specifically address whether and how our model maps to some of the different aspects reviewed by Cleeremans (2005) as are Access Consciousness and Metacognition. We will argue that the network studied does show some aspects of Access Consciousness, but not of Metacognition and will finish with Conclusions in Section “Discussion and Conclusion.”

## ReaCog, an embodied, minimal version of a cognitive system

The network reaCog represents an expansion of a neural network based controller called Walknet which has been derived as a hypothesis to describe a large number of behavioral studies performed with stick insects (Dürr et al., 2004; Schilling et al., submitted a).

**Figure 1:**
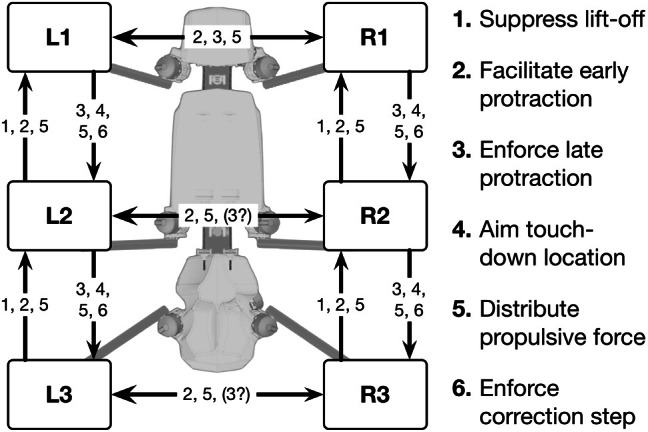
**Schema showing the robot Hector and the morphological arrangement of the leg controllers and the coordination influences (1–6) between legs**. Legs are marked by L for left legs and R for right legs and numbered from 1 to 3 for front, middle and hind legs, respectively.

**Figure 2:**
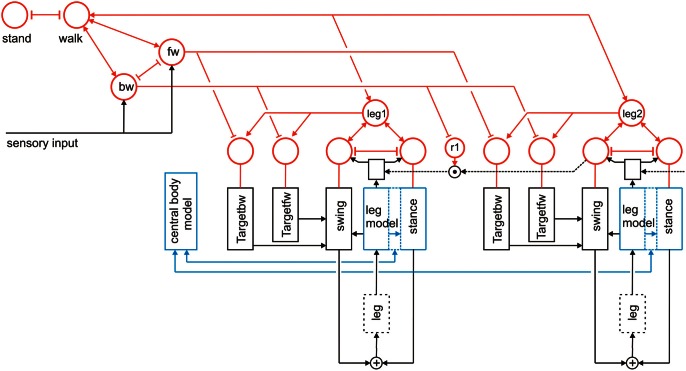
**A section of Walknet showing two leg controllers**. Each consists of a Stance-net and a Swing-net, the latter being connected with a Target-net (Targetfw). The motor output acts on the legs (box muscles/body/environment). Sensory feedback is used by the motor procedures as well as to switch between the states (red units connected by mutual inhibition). r1 represents coordination rule 1 (see Figure 1). Furthermore, the network is equipped with further procedures (Target_bw-net), a body model (blue) and a motivation unit network (red). The body is represented by the boxes “leg.”

The controller has to deal with a body containing 22 degrees of freedom (DoF), 3 DoF for each of the six legs and 4 DoF allow for movements along the body axis. As body position in space is defined by only 6 DoFs (three for position in space, three for orientation) there are 16 DoFs free to be decided upon. The controller consists of a decentralized architecture, first of all six more or less independent controllers, one for each leg. The controllers of neighboring legs are coupled via a small number of channels transmitting information concerning the actual state of that leg (e.g., swing, stance) or its position, i.e., values of joint angles (Figure 1). The architecture of the leg controller is depicted in Figure 2, lower part, black boxes. Only two leg controllers are shown. The single leg controller consists of several procedures that are realized by artificial neurons forming a local, in general, recurrent neural network (RNN). These procedural elements, or modules, might receive direct sensory input and provide output signals that can be used for driving motor elements. But other modules may also provide input to a module. All these networks may be considered to form elements of the procedural memory. The two most important procedural elements in our example are the Swing-net, responsible for controlling a swing movement, and the Stance-net controlling a stance movement. In addition, each leg possesses a so-called Target_fw-net for forward walking and Target_bw-net for backward walking, both influencing Swing-net.

To allow the system to select autonomously between different behaviors as for instance standing and walking, or forward and backward walking, reaCog is expanded by introduction of a RNN consisting of so-called motivation units (Figure 2, marked in red). The function of a motivation unit as applied here is to control to what extent the corresponding procedural element contributes to the behavior. To this end, these units influence the strength of the output of its procedure network (in a multiplicative way). As illustrated in Figure 2, motivation units can also be used to influence other motivation units via excitatory or inhibitory connections. For example, units which belong to the procedural nets controlling the six legs (only two legs are depicted in Figure 2) show mutual positive connections to a unit termed “walk” in Figure 2. This unit serves the function of arousing all units possibly required when the behavior walk is activated.

In addition, we introduce units “forward” and “backward” to activate procedures required for forward or backward walking (Figure 2, fw, bw), respectively, by selecting specific Target-nets. Both units “fw” and “bw” are mutually coupled with the motivation unit “walk.” Only indicated in this figure is that the unit “walk” may be coupled via mutual inhibition to other units that stand for different behaviors like, for example, standing still (unit “stand”). The corresponding procedures are, however, not depicted. It is also not shown that these “higher-level” motivation units may receive direct or indirect input from sensory units that influence the activation of a motivation unit. In Figure 2 this is only shown for the “lower-level” motivation units of Swing-net and Stance-net (for example, a ground contact sensor of a leg being stimulated may activate the motivation unit of the stance procedure of this leg). Also, the complete motivation unit network used for controlling navigation is not shown (see Hoinville et al., 2012).

As illustrated in Figure 2, this at first glance hierarchical structure of the motivation unit network is in general not forming a simple, tree-like arborization. As indicated by the bi-directional connections, motivation units form a RNN coupled by positive (arrowheads) and negative (T-shaped connections) influences (for details concerning the weights used see Schilling et al., submitted b). This structure may therefore be better described as “heterarchical.” Some of these motivation units are coupled by local winner-take-all connections. This is true for the Swing-net and Stance-net of each leg, as well as for the motivation units for forward and backward walking. Thereby, a selection of one of the available Target-nets is possible. Excitatory connections between motivation units allow for building coalitions. As can be derived from Figure 2, there are different overlapping ensembles possible. For example, all “leg” units and the unit “walk” are activated during backward walking and during forward walking, but only one of the two units termed “fw” (forward) and “bw” (backward) and only some of the targeting modules are active in either case. In this way, through the combination of excitatory and inhibitory connections this architecture can produce various stable attractor states or “internal states.” Such a state protects the system from responding to inappropriate sensory input. For instance, as a lower-level example, depending on whether a leg is in swing state or in stance state, a given sensory input can be treated differently. Correspondingly, internal states can be distinguished on higher-levels, as for example walking, standing still, or feeding (for further details see Schilling et al., submitted a,b).

### Body model

**Figure 3:**
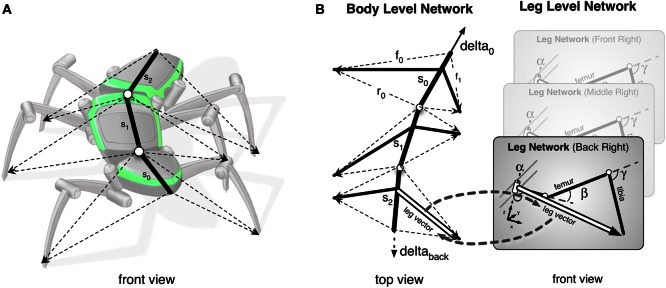
**(A)** Illustrates how the body model is attached to the body of robot Hector. **(B)** shows the abstracted body model. Vectors delta_0 and delta_back can pull the model in forward or backward direction, respectively. On the right, an example is shown how a leg network is connected to the abstracted/central body model.

A further important element of reaCog concerns the representation of a body model. This body model is realized by a specific RNN (Schilling, 2011) and has by itself a modular structure (Schilling and Cruse, 2007; Schilling et al., 2012). It consists of six networks each representing one leg. These modules are connected on a higher-level forming a seventh network representing the whole body. The latter network represents the central body and the legs in an only abstracted form. In Figure 2 the elements of the body model are marked in b1lue. Thus, the body model is represented by a modular structure which, as it is constructed as a RNN, at the same time comprises a holistic system [Figure 3, for details concerning the body model see (Schilling, 2011; Schilling and Cruse, 2012)].

In normal walking, i.e., still in the reactive mode, the body model is used in forward and backward walking as well as in negotiating curves and provides joint control signals to the corresponding Stance-net. As the model mirrors the 22 DoF of the insect body the task is underdetermined. Therefore, calculation of the joint control signals is still a hard problem and a unique solution is not directly computable (Schilling and Cruse, 2012). As a solution, we apply the idea of the passive motion paradigm to this problem (von Kleist, 1810; Mussa Ivaldi et al., 1988; Loeb, 2001). Like a simulated marionette puppet (Figure 3), the internally simulated body is pulled by its head in the direction of desired body movement (Figure 3B, delta_0), provided, for example, by a vector based on sensory input from the antennae or, if available, by visual or acoustic input (Figure 2, sensory input). As a consequence, the stance legs of the puppet move in an appropriate way. The changes of the simulated joint angles can be used as motor commands to control the actual joints. To control backward walking, the body model is pulled by the vector delta_back (Figure 3B) at the bottom. If such a body model is given that represents the kinematical constraints of the real body, we obtain in this way an easy solution of the inverse kinematic problem, i.e., a solution for the question how the joints of legs standing on the ground have to be moved in concert to propel the body.

The body model also receives sensory data. Due to its holistic structure the body model integrates redundant sensory information and is able to correct possible errors in the sensor data (Schilling and Cruse, 2012). As will be sketched below, due to its ability of pattern completion, this model can also be used as a forward model. Therefore, the model allows for prediction, too, a property that can be exploited when dealing with the ability to plan ahead.

### Planning ahead

The network, as described, consists of a “hard-wired” structure, i.e., the weights connecting the artificial neurons are fixed. Nevertheless, the system is able to flexibly adapt to properties of the environment, as for example deal with various disturbances and climb over large gaps (Bläsing, 2006). However, situations may occur in which the controller runs into a deadlock. Think for example of the situation in which, during forward walking, by chance all legs but the right hind leg are positioned in the frontal part of their corresponding range of movement, whilst the right hind leg is positioned very far to the rear. When this leg starts a swing movement, the body may fall backward as the center of gravity is not anymore supported by the legs on the ground. Such a “problem” might be signaled by specific sensory input, a “problem detector.” In our case, this could, for example, be a system reacting to a specific load distribution of the legs. To find a way out of this deadlock, a random selection of a behavioral module not belonging to the actual context could provide help. A possible solution in this case might be a backward step of the right middle leg. Such a backward step of the middle leg would make it possible to support the body, then allowing the hind leg to start a swing. However, in our controller, backward steps are only permitted in the context of backward walking. How might it still be possible for the system to find such a solution?

**Figure 4:**
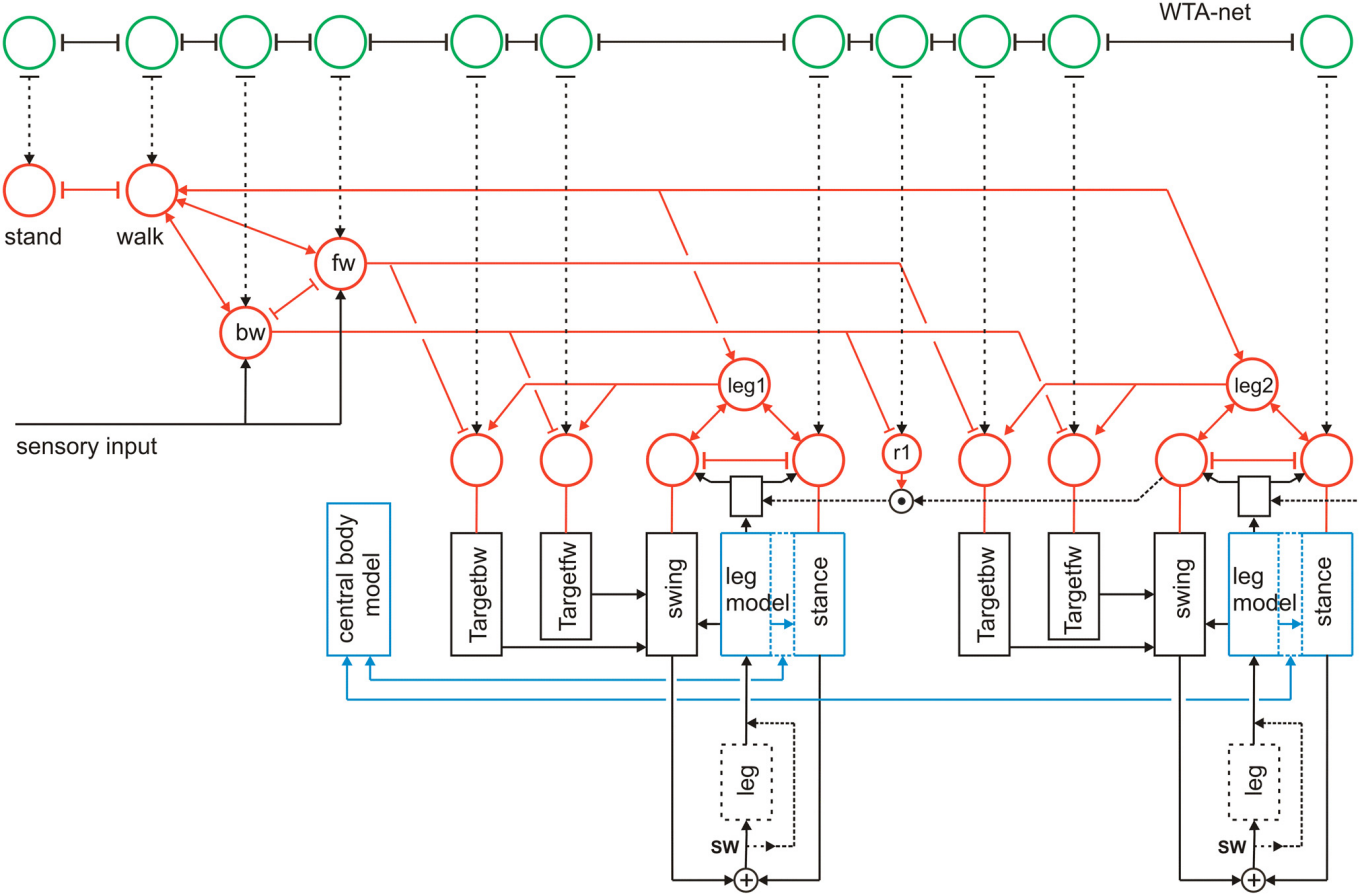
**Schema of reaCog, consisting of Walknet as depicted in Figure 2, with a body model (blue) and a motivation unit network (red), but now expanded by a further layer (WTA, marked green, not all connections are depicted)**. The body is represented by the boxes “leg.”

Figure 4 illustrates a simple expansion allowing the system to search for such a solution. As we will argue later, we name this expansion “attention controller.” A third layer (Figure 4, green units), essentially consisting of a recurrent winner-take-all network (WTA-net)^1^ is arranged in such a way that each motivation unit has a partner unit in the WTA-net. Motivation units already activated in the actual context inhibit their WTA partner unit (T-shaped connections in Figure 4). Thus, a random activation of the WTA-net will, after relaxation, find a unit not belonging to the currently activated modules. The WTA unit winning the competition can then be used to activate its partner motivation unit and thereby trigger a new behavior that can be tested for being able to solve the problem. In this way, the network has the capability of following a trial-and-error strategy.

As has been proposed (Schilling and Cruse, 2008) a further expansion of the system may permit to use the body model instead of the real body to test the new behavior via “internal trial-and-error” whilst the motor output to the real body is switched off. To this end, switches have to be introduced allowing the motor output signals to circumvent the real body and being passed directly to the body model (Figure 4, switch SW). Only if the internal simulation has shown that the new trial provides a solution to the problem, the behavior will actually be executed. McFarland and Bösser (1993) define a cognitive system in the strict sense as a system that is able to plan ahead, i.e., to perform internal simulations to predict the possible outcome of a behavior. Therefore, the latter expansion would, according to McFarland and Bösser, make the system a cognitive one (for details see Cruse and Schilling, 2010; Schilling and Cruse, submitted).

### General characteristics of reaCog and a possible expansion

To sum up, the neural controller Walknet, as described earlier (e.g., Dürr et al., 2004; Schilling et al., submitted a), represents a typical case of an embodied controller (first-order embodiment, cf. Metzinger, 2006, forthcoming): the network is able to control the movement of a hexapod walker in unpredictably varying environments without relying on other information than available using the given mechanosensors. This is possible because the body and properties of the environment are crucial elements of the computational system – the system is embodied not only in the sense that there is a physical body (e.g., that there are internal states being physically represented), but also in the sense that the properties of the body (e.g., its geometry) are required for computational purposes. Exploiting the loop through the world (including the own body) allows for a dramatic simplification of the computation. These properties can also be attributed to the expanded version, reaCog. In this system, being expanded by an internal body model, control of DoFs does not result from explicit specification by the neuronal controller, but results from a combination/cooperation of the neuronal controller, the internal body model and the coupling via the environment. Furthermore, the body model is used for planning ahead. Such a network, according to Metzinger (2006, forthcoming), represents a system being characterized by second-order embodiment.

The procedures forming the decentralized controller are basically arranged in parallel, i.e., obtain sensory input and provide motor output, but there are also procedures that receive input from other procedures and, as a consequence, procedures that provide output to other procedures.

The artificial neural network reaCog shows automatic behavior and action selection on the reactive level, where several of these procedures can be performed in parallel, but also shows control of behavior on the cognitive level, as the decisions based on imagined action (probehandeln) are not determined strictly by the sensorily given situation. This is the case because due to the noise active in the attention controller, there is a stochastic effect. The final decision is, however, not purely stochastic, because the proposals made by the attention controller are tested for feasibility via the internal simulation. Before being stored in long term memory, the proposal is further tested by performing the behavior in reality. In this way, this decision may be viewed as to be based on a Darwinian procedure, starting with an, in part, stochastic “mutation,” followed by an, in our case twofold, selection testing the proposal for “fitness.”

**Figure 5:**
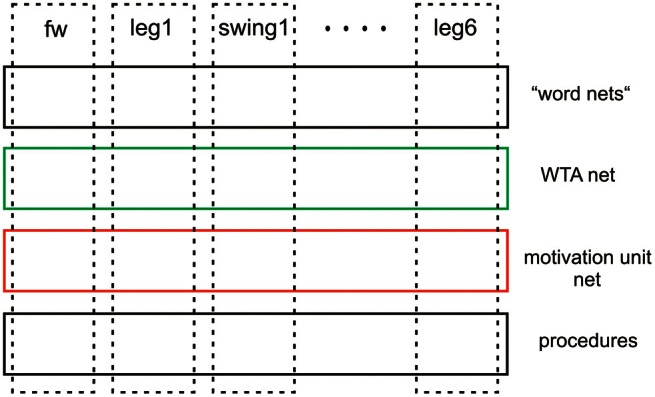
**Schematic showing the horizontal and columnar arrangement of the modules used by the architecture proposed**.

Furthermore, inspired by Steels (2007); Steels and Belpaeme (2005), the network may be expanded by a forth layer (not depicted in Figure 4), that contains specific procedures, namely networks that represent verbal expressions. These “word-nets” may likewise be used to utter or to comprehend the word stored. The underlying idea is to connect each word-net with a unit of the motivation network of which it carries the meaning (e.g., the word-net “walk” should be connected with the motivation unit walk), thereby grounding the symbolic expression (Cruse, 2010). Although the latter two levels (WTA-net and word-nets) are still quite speculative as they have not yet been tested, together with the two lower layers they illustrate the principal idea of this architecture (Figure 5). Horizontally arranged modules (procedures, motivation units, WTA neurons, and procedures for words), are ordered in the horizontal layers in such a way that the corresponding elements in the different layers appear in a vertical order, leading to modules arranged in a columnar fashion (Figure 5, dashed rectangles). Addressing this columnar structure does not mean that each lower-level procedure or each motivation unit has to have a partner in the upper layers, but only means that such connections are in principle possible. Similarly, not every unit or procedure in the upper layers necessarily has a partner procedure in the lowest layer.

## Properties of reaCog being characterized by applying other levels of description

Having available a quantitatively defined network that is able to control specific behaviors of an agent, we will now ask to what extent reaCog is able to realize properties that were not explicitly implemented. For example, as has been noted earlier (Schilling et al., 2008, submitted a), when applying descriptions used in the behavioral domain, a term like tripod gait is sensibly used to describe the walking behavior of a hexapod, although no explicit tripod gait controller, for instance, is implemented in reaCog (different to many other hexapod controllers). Instead, at the neuronal/computational level only local rules are used to couple neighboring legs, which allows for different walking patterns to appear, depending on the control parameter “velocity.”

In the following, we will particularly concentrate on concepts usually applied in domains other than computer science and behavioral biology, as are psychology and philosophy of mind. Adopting other levels of description may not only be feasible to better understand the properties of our system on a more abstract level, but may also help to find more operational definitions for concepts used in the other disciplines. Underlying such an approach is the assumption that most, if not all of these phenomena arise as emergent properties (Vision, 2011) and that they can only be observed and characterized when higher-levels of description are applied.

Whereas some authors speculate that phenomena as for instance consciousness can only be attributed to human beings or possibly monkeys, other authors claim that consciousness may come in various degrees and may, to a smaller degree, already occur in lower-level animals (Dennett, 1991). This view is supported by the observation that already small-scale networks might allow for interesting cognitive properties (Herzog et al., 2007; Menzel et al., 2007). Due to its evolutionary plausibility we tend to the latter assumption, and therefore raise the question to what extent any aspects of consciousness could be attributed to the network discussed here although, when designing the network, we did not aim to “implement consciousness” at all. To the extent such attributions would be possible, questions concerning the possible function of consciousness, for example as to how consciousness might contribute to action, i.e., to the control of behavior, might be addressable.

We would like to stress that, in pursuing this question, we are not trying to state what consciousness is, i.e., we do not want to “explain consciousness.” We also do not assume that the categories introduced by the different authors referred to represent the ultimate solution to approach the problem. Instead, we would like to connect aspects of this complex issue, as have been addressed by different authors, with our simulation approach. This further means that the collection of properties characterizing a system as being conscious will not be discussed in a rigorous way with respect to being necessary or sufficient for a system being a conscious one. Rather, we will only compare the categories discussed by different authors with our approach. A rigorous definition might only be sensible at a later stage (see also Holland and Goodman, 2003).

To this end, we will begin by following a categorization proposed by Block (1995, 2001) and being placed in a broader framework by Cleeremans (2005). Cleeremans reviewed an impressive number of philosophical statements concerning consciousness. In spite of considerable disagreement between authors in detail (see also Vision, 2011), Cleeremans reported an interesting overlap with respect to the essential properties characterizing possible computational correlates of consciousness. According to this review, phenomena concerning consciousness may be grouped along three domains, termed Phenomenal Consciousness, Access Consciousness, and Metacognition (or Reflexive Consciousness). Concerning the phenomenal aspect of consciousness, some philosophers consider this aspect as a separate domain, being independent of Metacognition and Access Consciousness, whereas other philosophers consider phenomenality as a property not being separable, but being directly connected with Metacognition and Access Consciousness. Again, other philosophers are only prepared to attribute consciousness to systems showing Reflexive Consciousness (e.g., Rosenthal, 2002). As mentioned, we will not enter this discussion. For our purpose it is not critical which of the different taxonomies is better suited to characterize the phenomenon of consciousness. We selected one, Block's taxonomy, as a scaffold to compare the different phenomena described in the literature with properties of our network.

### Phenomenality

What is meant by phenomenal consciousness or the phenomenal aspect of consciousness, sometimes also termed internal perspective or subjective experience? The characteristic of subjective experience may be particularly obvious in the case of pain. We might, as a thought experiment, monitor all neuronal activities of a (human) subject that result when his/her skin is stimulated by a needle. One might, in principle even examine one's own action potentials, if oneself is the subject of this experiment. In such an experiment, everybody including the subject him/herself could have a look at the data, but the experience when regarding all these neuronal activities monitored is completely different from the pain one is experiencing at this moment. The content of this subjective experience is only accessible to the person herself or himself. Nobody other than myself can judge how I feel the pain. Thus, self-observation tells us that there are systems, namely humans, that can experience an internal perspective. On the other hand, intuition tells us that there are other systems, like a stone or a simple machine (including some clever present-day robots) that may not have such an internal perspective.

In many cases, consider for example an animal like an insect, we cannot decide whether it belongs to systems that act like a reflex machine, or a clockwork, not being able to experience an internal perspective, or whether it belongs to the second type, and consequently is able to have subjective experience.

But also within the human brain there are sections that belong to one of both states and that may even be able to switch between both states. In (dreamless) sleep or under anesthesia neuronal systems are still active but subjective experience is “switched off.” But also when in normal awake state, we are not aware of the contents of all the different neuronal activities taking place in our brain. Rather, at a given moment we consciously attend, and therefore subjectively experience, only one aspect and may later switch conscious attention to another one. Therefore, we have to assume that subjective experiences arise only if specific, yet unknown, types of neuronal activities are given.

Up to now we have only indirect evidence concerning the conditions required for subjective experience to arise. In an early experiment, Libet et al. (1964) performing direct electrical stimulation of the cortex found that a stimulus required a minimum of 500 ms to lead to a reportable experience. More generally, according to Bloch's law (Bloch, 1885), the subjectively experienced strength of a stimulus depends on the mathematical product of stimulus duration and stimulus intensity. This means, in other words, that the temporal integral over stimulus intensity has to reach a given threshold to become subjectively experienced.

In more recent experiments, activation of different procedures have been studied which compete for becoming subjectively experienced. For example, Ansorge et al. (1998) performed masking experiments, where participants first learned to press a button when a circle was presented on a screen, but not when a square was shown. After learning is finished, in the critical experiment the circle was given for a short period (about 30 ms) which was then followed by a longer presentation of the square. The participants reported to have only seen the square. Nonetheless, they pressed the button. This result can be interpreted in such a way that the procedure, “stimulus circle-motor response” can be executed without being accompanied by subjective experience of the circle. The second procedure, “stimulus square – no motor response” apparently influences the first procedure by inhibiting the process leading to subjective experience. This is interpreted in the following way: each procedure shows a temporal dynamics similar to that of a low-pass filter^2^. The motor command of a procedure can already be elicited after a smaller threshold has been reached, whereas a larger threshold is required to reach the state of subjective experience. Only in the latter state the procedure can inhibit other, competing procedures to reach the state of subjective experience. In other words, procedures appear to be connected via a WTA network, where the inhibitory connections are only active when the procedural network has reached the (higher) threshold characterizing the state of subjective experience. Therefore, in the masking experiment the second procedure is not inhibited by the first one, which allows the square to become subjectively experienced.

These results lead us to the following view. There are specific neuronal states that require time to be developed. The basic function of the neural system, namely triggering the output signal (e.g., a motor command) can be performed without phenomenal experience, but at least some procedures may in addition be able to reach the latter state. After the neural network has reached this state, additional functions may arise, one, as mentioned, being to inhibit other procedures to reach this state. Other functions might be to allow the winning procedure to access more neuronal sources, and perhaps to allow faster storing of new information (e.g., for one-shot learning).

It would of course be extremely interesting to understand in detail the conditions that are necessary and sufficient for a neuronal network to reach the state being accompanied by subjective experience. At this time merely pure speculations are possible concerning the character of such neuronal activities although impressive progress has been made in recent years (see review Schier, 2009; Dehaene and Changeux, 2011). Continuation of these research projects by combining neurophysiological with behavioral studies may lead to a better understanding of the physiological properties and functions of this state. But even if this was the case at some future time, we would not understand *why* this state is accompanied by the phenomenal aspect.

The results mentioned above support a non-dualist, or monist, view, which means that there are no separate domains, the mental and the physical domain in the sense that there are causal influences from one domain to the other one as postulated by substance dualism. Rather, both “domains” appear to be different aspects of the same underlying phenomenon. We just deal with different levels of description^3^.

Adopting a monist view allows us to concentrate on the functional aspects when comparing systems endowed with the phenomenal aspect, i.e., human beings, with animals or artificial systems. According to this view, phenomenality is considered a property being directly connected with specific functions of the network. This means that mental phenomena that are characterized by phenomenal content as are, for example, attention, intention, volition, emotion, and consciousness, can be treated by concentrating on the aspect of information processing (Neisser, 1967). In particular with respect to Phenomenal Consciousness, Access Consciousness, and Metacognition, this view has convincingly been supported by Kouider et al. (2010) as well as, in a recent review, by Cohen and Dennett (2011). Therefore, we will compare properties of reaCog with current definitions found in the literature concerning those phenomena. In doing so, we have however to be aware of the possibility that important functional properties may not yet be taken into account by these definitions.

Following the monist view, the question as to how it is possible that a physical system is accompanied by subjective experiences, termed the “hard problem” by Chalmers (1996), can remain open and we may yet be able to understand the functional aspects of consciousness. A further consequence would be that even an artificial system would have some kind of subjective experience, if only the appropriate (yet unknown) neural dynamics were implemented (for ethical problems connected with this matter see Metzinger, 2009). On the other hand, it might be possible that systems exist where the functional aspects currently attributed to consciousness are given although these systems are not accompanied with phenomenality, because the networks show the functions of phenomena as listed in the following sections but do not show the neural dynamics required for phenomenality. In the following, we will first address briefly attention, volition and intention, and then deal with consciousness.

### Attention, volition, intention

Can we find properties corresponding to attention in reaCog? Attention concerns how perception is selected by bottom-up, i.e., sensory driven influences, or by top-down influences (Desimone and Duncan, 1995). The latter may depend on familiarity with the stimulus, or on internal (e.g., emotional) states. Concerning reaCog, there are, indeed, several cases to be observed.

The motivation unit network is especially designed to allow for competitions on different levels, in this way forming different clusters, or coalitions, of units. For example, the competition on a leg level selects between swing and stance movements. Stimulation by the ground contact sensor, for instance, changes the internal state from swing to stance. Activating the unit Stance means that sensory input relevant for stance, but not inputs relevant for swing can be perceived. Therefore, this case corresponds to bottom-up attention control.

On a more global level, behaviors different from walking or, within the context of walking, the direction on forward or backward, can be selected. Activation of these motivation units not only allows for selection of behavioral elements, but also provides a broader context according to which specific sensory inputs may be selected or not. In this sense, the motivation unit network can be considered to be a system allowing for top-down attention control. In the case of Navinet, for example, visual signals are only considered when they belong to the currently activated context defined by looking for a specific food source. The context might be changed when the food source is found to be empty.

Introduction of the cognitive expansion enables reaCog to invent new behaviors and to test them via internal simulation before executing them. In this layer, the WTA units of the cognitive expansion are arranged in accord with the motivation units in the lower layer. As this expansion of the reactive network allows the complete system, using psychological terms to describe its function, to “focus” or “concentrate” or “attend” on a specific behavior, we may also call this expansion an “attention controller^4^.”

This system represents a special type of top-down attention being used to select new procedures, normally not used in the current context. The decision to execute a new behavior as controlled by the attention controller will be called a cognitive decision in the following. This focusing mechanism may correspond to what sometimes has been termed “spot light” (Baars and Franklin, 2007). Thus, three types of attentional influences can be observed in reaCog. If the procedures controlled by the motivation units are equipped with the still unknown neural dynamics required for phenomenality, their content could reach the state of subjective experience.

Volition is a summary term denoting mechanisms allowing for voluntary actions. The latter are “actions that are not fully determined by the immediate stimulus situation but depend on mental representations of intended goals and anticipated effects” (Goschke, 2013). In other words, the behavior of the agent cannot be predicted by an external observer. Cognitive decisions made by reaCog are indeed based on anticipated effect using internal simulation and they follow a goal, as they aim to solve the problem at hand. These decisions contain a stochastic element, but are not arbitrary because the proposed behavior is tested via internal simulation for feasibility before being executed and because the architecture of the WTA-net being connected to the body already represents a heuristic based on some kind of topological map (solutions near the morphological site of the problem are supported). Therefore, volition may be attributed to an agent controlled by reaCog, whereby, as above, the phenomenal aspect depends on the unknown conditions concerning the required neural dynamics.

Similarly, an agent controlled by reaCog might be attributed the capability of showing intentions. An action is controlled by intention if it is goal-directed. Pacherie (2006) referring to Bratman (1987) distinguishes between different types of intentions based on the temporal characteristics: future-directed intentions and present-directed intentions. Pacherie (2006) adds a third type, called motor-intentions. The latter two are characterized as to guide either “higher-level” functions or “lower-lever” functions, respectively. According to Pacherie (2006), present-directed intentions, in contrast to motor-intentions, are considered as under “conscious” control or “rational” control. In our framework, we interpret this in such a way that motor-intentions act on the reactive level, whereas present-directed intentions require cognitive decisions. Future-directed intentions concerning long term planning are not considered here. In any case, the basic underlying control structure is given by a feedback controller and/or by a feedforward controller containing explicit or implicit representations of the goal. However, the actual behavior may require a network for the control of many more parameters including temporal aspects s is the case in reaCog. According to Goschke (2013), intentions are “causal preconditions explaining why a particular stimulus triggers a particular action (rather than a different action)” (Goschke, 2013, p. 415) In other words, “intentions can be said to shape the “attractor landscape” of an agent's behavioral state space” (Kugler et al., 1990, ref. from Goschke, 2013, p. 415). Indeed, the motivation unit network is able to form such attractor states, for example, when in Navinet the agent has decided to visit a specific food source or the nest. Depending on the actual goal, the relevant behavior will be executed while specific sensory stimuli are attended or not. Therefore, the agent may be called to be endowed with intentions.

### Access consciousness

As mentioned earlier, our approach is *not* to start with theoretical concepts of consciousness (or attention) and then construct a network that is endowed with properties of consciousness. In contrast, our goal is to construct a network that, based on a reactive network, is able to control non-trivial reactive behavior, and shows cognitive abilities, i.e., is able to invent new solutions for a problem and to plan ahead. Only after having such a system available, we ask whether it may also be attributed with properties related with consciousness. Specifically, as we abstract from the phenomenal aspect, we will refer to Access Consciousness and Metacognition.

To begin with, we will focus on the question whether, in reaCog, we would find properties of Access Consciousness. This question would be of interest even if some authors would be correct who argue that consciousness in the strict sense can only arise in systems showing the faculty of Metacognition (e.g., Lau and Rosenthal, 2011, for a recent review defending this view).

The essential properties of Access Consciousness (e.g., Cleeremans, 2005) refer to the ability of a system to plan and guide actions, to report verbally on the content of the corresponding representations and to reason. In contrast, non-conscious representations cannot be used this way. As discussed in Section “Planning Ahead,” planning ahead and guiding actions are indeed central properties of reaCog. An agent equipped with reaCog is able to, first, test a new idea by internal simulation (“probehandeln”), which will, when the test has been successful, then be used to guide the newly invented behavior. Concerning the third issue of Cleeremans's list, verbal report, we only briefly sketched here how reaCog may be equipped with the property to deal with (verbal) symbols allowing the agent to report on internal states and comprehend heard verbal expressions (Cruse, 2010). Steels (2007), Steels and Belpaeme (2005), and Narayanan (1997) have however studied in much detail how these properties may be incorporated in a network being based on reactive structures. Thus, at least in principle, reaCog could realize this property, too. Only the last issue from this list describing properties of Access Consciousness, symbolic reasoning, is clearly not addressed by reaCog.

#### Related work

To illustrate in more detail to what extent reaCog shows properties of Access Consciousness, we compare reaCog with other related approaches. Dehaene and Changeux (2011) review the relevant models of networks that are supposed to simulate consciousness, including their own approach “global neural workspace” (GNW) (see also Seth, 2007 for a systematic summary). Of all models discussed by Dehaene and Changeux, GNW shows the largest overlap with reaCog. Therefore, in the following we will focus on a comparison with this approach.

Following the ideas of Baars and colleagues (e.g., Baars, 1988; Baars and Franklin, 2007), who, starting with an abstract conceptual approach, have developed the “global workspace” theory, Dehaene and Changeux continued these ideas developing a neural implementation of the GNW. Coarsely, this model consists of two parts, a number of specialized, automatic processes, considered non-conscious, and a second, upper-level part, to which properties of consciousness are attributed. The function of this “router” is to connect sensory and motor representations by variably connecting different automatic processes. Thereby, this “router” is responsible for amplifying and maintaining specific neural representations, making them consciously accessible. Due to the long distance connections the content of these representations can be globally “broadcasted” to many other processes in the brain.

Let us begin to address the basic differences between the GNW model and reaCog. The first one concerns the architectural details, in particular the granularity of the models. GNW operates with a large number of spiking neurons (two orders of magnitude more neurons than reaCog) simulating in detail membrane properties, ion channels and receptor potentials, like AMPA or NMDA receptors. In reaCog only very simple, piecewise linear, weighted summation units are used.

The GNW model consists of several layers connected via bottom-up and top-down channels. Elements of the uppermost layer are connected via mutual inhibition which leads to a competition between these elements (like in a WTA-net). A weak and/or short stimulus given to the lowest, input, layer elicits a short, decaying excitation of the upper layers. A strong and/or long stimulus may activate the top-down connections in such a way that long reverberating activity will occur showing long range synchronous oscillations. The former case is compared with non-conscious activity, the latter with conscious activity (In humans the latter is paralleled by specific oscillations in the gamma band and also marked by positive waves in event-related potentials, Dehaene and Changeux, 2011). As the elements of the uppermost layer, the “router,” compete with each other, only one of these elements can be active (and reach the conscious state) at a given moment of time, whereas weaker stimulation may activate several lower-level elements in parallel, maintaining them in the non-conscious state.

In reaCog, we have only one layer of procedures the activity of which could realize different internal states. These states correspond to different contexts that can control the automatic, non-conscious behaviors. If a problem occurs, the attention controller selects and activates specific lower-level procedures by activating the corresponding motivation units which may form coalitions. Like in the upper layer of GNW, there is a competition based on lateral inhibition represented by the “attention controller,” i.e., essentially the WTA-net. Thus, both models allow for serial (all or none) processing at this level. The event-related potentials, in humans paralleled with the occurrence of subjective experience, could by both approaches be explained by the strong activation of the inhibitory signals used for competition in the uppermost layer of the GNW model and the WTA-net in reaCog. Both models further agree with the requirement (Cleeremans, 2005) that in order to reach Consciousness a high strength of activation is needed to win the WTA competition. Furthermore, some time is required as in both models several iterations are necessary until a unique decision has been made. Therefore, access to these attended elements, represented by the upper layer in the GNW model or the WTA units of the attention controller in reaCog, is slower than the reactive or “automatic” activation of a module remaining in unattended state. An essential difference between both approaches is that, in reaCog, this WTA-net does not contribute to the phenomenal state directly, but only selects those procedures that may become conscious. In reaCog, phenomenal experience, if given at all, is accompanied with the corresponding activation of the procedures.

Beside the difference with respect to granularity, the second crucial difference concerns the tasks to be dealt with. The task of the reactive part of reaCog is to control a complex body with 22 DoF – most of which concern redundant DoFs – able to walk over irregular surfaces including very large gaps (up to twice the size of a normal step length) as well as dealing with complex navigation tasks including path integration and landmark navigation. This is different from the GNW approach. A recent implementation of the GNW model, merging elements that have earlier been studied separately, is given by Zylberberg et al. (2011). The GNW model is equipped with the above mentioned complex internal neuronal structure forming a realistic simulation of mammalian brain properties. As input, simulated visual or auditory signals are applied whereas motor outputs are represented by simple go-nogo signals. As studied by Zylberberg et al. (2011), the GNW model concentrates on dual-task inferences, i.e., the inability of human subjects to deal with two tasks, T1 and T2, at the same time. In one type of experiments, studying the psychological refractory period (PRP), first a stimulus S1 is given, that triggers a task T1. Then a second stimulus, S2, triggering another task, T2, is provided. If S2 is presented before T1 has been finished, the execution of T2 is delayed until the first task is finished. In another type, the attentional blink experiment, a stimulus does not become consciously aware if it follows another stimulus too closely. In some masking experiments, the first stimulus is responded to although the person did not become aware of the appearance of this stimulus (e.g., Ansorge et al., 1998). The model of Zylberberg et al. (2011) is able to agree in quantitative detail with many experimental results. Generally, these effects can be interpreted as basic properties of a WTA network with hysteresis properties, the effects depending on the time delay, the strength, and the duration of the stimuli. Therefore qualitatively they could also be found in a network like reaCog. However, no comparable quantitative simulation is possible due to the different granularity. Likewise, no statements can be drawn from reaCog simulations which are comparable with the interesting insights (Zylberberg et al., 2011) concerning the possible properties of oscillatory states.

As reaCog is not equipped with spiking neurons, no long distance phase synchrony can be observed. These events are sometimes assumed as to form the neural correlates of consciousness. As an alternative, they may, however, as such, be mere “technical” requirements necessary for binding of spatially distributed neural elements, a function that in reaCog is represented by selection of the appropriate motivation units.

#### Global accessibility

A notion tightly related to the above mentioned term “GNW,” (e.g., Dehaene and Changeux, 2011), the term “unified neural workspace” (Dehaene and Naccache, 2001), and “global workspace” (Baars and Franklin, 2007), all postulated to characterize a prerequisite of conscious representations, concerns the latter as being “globally accessible” or “globally available” (Cleeremans, 2005). This means that many (but probably not all) of the representations stored in memory can become conscious representations, i.e., become available to be used for the solution of an actual problem (reaCog) or to be selected for a task (GNW). In contrast, nonconsciously used representations can only be used within their respective context.

To what extent can this aspect be represented by reaCog? If the agent is performing an automatic behavior, in this case walking on a not too strongly cluttered surface, the behavior can be driven by direct (and therefore fast) application of local modules belonging to the procedural memory. This is possible as long as no problem occurs. In such situations, the WTA-net of the attention controller is not activated which means that these behaviors are performed, but not “cognitively attended.” Therefore, the procedures are activated but not element of Access Consciousness as they are not used for planning, for example. However, when a problem happens to occur, most elements of the procedural memory can, in principle, be accessed by the attention system (Norman and Shallice, 1986). In reaCog this refers to those procedural elements that receive an influence from the WTA units (Figure 4, dashed arrows). Recall that, due to the properties of the WTA-net, only one such element can be activated at a given moment of time. All these modules may therefore be described as being “globally accessible” and possible elements of Access Consciousness.

#### Relation between conscious and automatic procedures

There is another interesting relation between properties of reaCog and findings in psychology, but has, to our knowledge not yet been addressed by the GNW approach. On a qualitative level it is known for long that we can learn new behaviors by treating them consciously, but with time of practice we are able to perform these behaviors more and more without conscious awareness being necessary (sometimes dubbed “downloading into the amphibian brain”). A similar process can be observed to happen in reaCog: as long as learning a new solution has not yet reached a level where no significant errors occur, the problem detectors are still active and the corresponding behavior remains attended. If learning was successful, attention is not any more necessary and the new solution has become part of the procedural memory, i.e., of the reactive system^5^.

On the other hand, there are experimental results showing that, in human beings, conscious access to an element after learning has been finished may lead to problems. Beilock et al. (2002) have shown that well-trained athletes perform better when they are distracted from the task than when they concentrate on performing a well-trained behavior. In principle, this property could be found in reaCog, too. If a WTA unit of the attention controller is activated by any higher-level brain structures (not addressed in Figure 4), this influence may activate learning and therefore change, and possibly deteriorate, the properties of the neuronal module. If no such attention influence is active, the behavior may be performed in a perfect way.

#### Localizing access consciousness

Finally, another difference between the simulation studies of, on the one hand, Dehaene and colleagues and Baars and colleagues and, on the other hand, reaCog, should be addressed. Whereas in the former approaches activities accompanied with consciousness are assigned to specific areas of the human brain, we stay neutral with respect to analogies between the structures of reaCog and the morphology of the human brain due to our extreme reduction to function. Instead, we could ask whether it would be possible to localize the properties of Access Consciousness anywhere within reaCog? Interestingly, there is no specific part that might be attributed the property of Access Consciousness. Rather, the complete system consisting of procedural memory, the attention controller, and its ability to switch the motor output from controlling the body to controlling the body model, can be considered to correspond to the structure required for Access Consciousness or the “neural workspace.” Its dynamics, as defined by Dehaene and Naccache (2001), is, in the model, essentially determined by the dynamics of the WTA-net. In our model the neural workspace does not form a separate “theater” where the content of the memory elements is re-represented. Instead, already existing modules of the procedural memory being coupled via the loop through the model of the body and of the environment together form the global workspace (which compares to the notion of “second-order embodiment,” c.f. (Metzinger, forthcoming). reaCog is neither hierarchically structured nor is it strictly parallel, as the attention controller only selects the relevant processes. Therefore, reaCog should not be interpreted as a first-order model as defined by Lau and Rosenthal (2011), because the upper layer, the attention controller, is necessarily required.

#### Attention and consciousness

Koch and Tsuchiya (2007) argue that there is attention without consciousness and consciousness without concurrent attention, which leads these authors to the conclusion that both phenomena result from different mechanisms. This statement, of course, depends on how attention and consciousness are defined. If we accept a hypothesis for phenomenal experience to be based on specific neuronal dynamics, and the proposal made by reaCog that a stimulus is attended if specific motivation units are activated, in reaCog both phenomena are, although functionally related, indeed subject to different mechanisms. Attention refers to the selection of the procedure, which may reach a conscious state if attended for long enough time.

### Metacognition

The second, according Block (1995, 2001) and Cleeremans (2005), essential domain of consciousness, Metacognition, or Reflexive Consciousness (sometimes called Metarepresentation), is characterized by Lau and Rosenthal (2011) as “cognition that is about another cognitive process as opposed to about objects in the world^6^.”

Thus, when focusing on phenomenality, Metacognition can be described as referring to our ability not only to experience, but also to experience that we are experiencing. Correspondingly, when focusing on the execution of behavior, Metacognition refers to the ability of the metacognitive agent to select procedures to control behavior and, by doing so, representing himself or herself (“I make the decision”). In other words, Metacognition requires the ability to observe the own internal states from “above,” or “from a bird's eye perspective.” Metzinger (forthcoming) classifies this ability as third order embodiment, where the own body is “explicitly represented as existing” and the “body as a whole” can turn “into an object of self-directed attention.” Cognitive systems like reaCog can mentally manipulate only objects of the world, including parts of their own body. These objects are manipulated relative to themselves, i.e., in an egocentric world. In contrast, a metacognitive system can, in addition, manipulate a representation of itself relative to the other objects. In other words, metacognitive systems can consider themselves as an object of the world, an ability which may be described as allowing for an allocentric view. reaCog is not equipped with this ability, i.e., reaCog is not equipped with Metacognition.

On a more detailed level, a metacognitive system is characterized by being able to exploit information concerning the quality of the procedure, for instance when selecting a procedure to control the behavior. A person may, for example, access their internal states and guess to what extent he or she is sure about a specific memory content, in order to use this knowledge for decision making. Exploiting stored confidence values is, as such, also possible for a system like reaCog, for example, when the activation of a motivation unit depends on a confidence or quality value. This is indeed the case for the network Navinet mentioned above, which is able to control ant-like navigation allowing for decisions on memory retrieval which depend on the salience of the stored stimulus (Cruse and Wehner, 2011; Hoinville et al., 2012). However, reaCog, extended by Navinet, is not able to represent itself as an element that is mentally manipulable as are other objects of the world, for example its legs. Cleeremans et al. (2007) describe an artificial neural network consisting of two networks. One, a first-order network, learns a specific input-output task, whereas the other, second-order network learns to estimate the quality of the performance of the first network. The authors claim this system to show a limited form of Metarepresentation, because it represents not only knowledge *in* the system, but also knowledge *for* the system. Although being a very interesting result, we are hesitating to attribute such a system Metacognition as it lacks, like reaCog, a representation of itself.

## Discussion and conclusion

Thus, as a short summary, some of the properties attributed to Access Consciousness can be found in our network, at least in a basic form. Clearly missing are the ability of linguistic reasoning, whereas introduction of verbal communication is only sketched. reaCog may therefore be considered a system that could provide a scaffold for a later system being able to cover some basic aspects of consciousness concerning both Access Consciousness and, as addressed above, Metacognition as long as we put aside the subjective aspect.

The question as to whether it is allowed after all to apply the term consciousness, but also terms as attention, volition, intention (and, not addressed here, emotion) to a simple, insect-based artificial system could be answered in two ways: either these terms are defined as to be strictly coupled to a system that is known to be endowed with an internal perspective. Then, according to current knowledge, these terms are only applicable to human beings, because only in this case we have direct evidence for phenomenality to exist. If we, however, leave this condition open, we have to focus on the functional aspect, and search for corresponding properties also in systems other than human beings including artificial systems. This approach is possible because we believe that the phenomenal aspect is always coupled to specific, yet unknown, properties of the neuronal system which, at the same time, has functional effects and shows subjective experience. In other words, adopting a monist view, we assume that we can circumvent the “hard” problem, i.e., the question concerning the subjective aspect of mental phenomena without losing information concerning the possible function. Of course, we are not in a position to claim which of these structures, if any, are accompanied with phenomenality. If, however, the function of the, for example artificial system, would indeed correspond well enough to those of the neuronal structures that are accompanied with phenomenality, the artificial system may have this property, too.

Following these arguments, we have presented a network that is based on a decentralized architecture consisting of procedural, or reactive, elements. The reactive network consisting of two subnets, Walknet and Navinet, characterized by a heterarchical structure, allows for selection of different behaviors, which includes protection against, in the current behavioral context, non-relevant sensory input, thus representing a kind of implicit attention control. As a next “evolutionary” step the network is equipped with a flexible internal body model allowing for internal simulation of behaviors. Together with the introduction of an attention controller, the complete network, termed reaCog, comprises the ability to plan ahead and to invent new behaviors in order to solve problems for which no solution is actually available. This capability allows the system to test possible adaptations of behavior by internal simulation before carrying them out in reality. In this way the system may circumvent hazardous situations. As such, this attention system cannot function by itself, but only, like a parasite, operates on top of the reactive structures. Following the definition of McFarland and Bösser (1993), the network, being based on reactive procedures and being capable of planning ahead, can be termed a *cog*nitive system, giving rise to its name reaCog.

The architecture applied here integrates often discussed properties postulated to exist in neuronal systems, as are modularity, heterarchy, redundancy, cross modal influences (e.g., path integration and landmark navigation in Navinet), bottom-up and top-down attention control, i.e., selection of relevant input data establishing priorities, as well as application of internal models for prediction. The heterarchical structure used in reaCog comprises a simple realization of “neural reuse” as proposed in Anderson's (2010) massive redeployment hypothesis (2010). Due to the fact that some central structures as the motivation unit network and the body model are realized as a RNN, the complete network forms a holistic system.

This architecture provides an example showing that functional concatenation of modules required for the control of complex behavior does not necessarily require explicit coding, but may emerge from local rules and the coupling through the environment. The latter is illustrated by implementing the network in a, as a first step, dynamic simulation of a 2 DoF, wheeled robot (Navinet) and a 22 DoF hexapod robot. In a second step, its capabilities will be tested on the physical robot Hector (Schneider et al., 2011).

In this article, we particularly focus on the question to what extent aspects of consciousness may be attributed to this system and in which way consciousness may allow for the control of action? Following Block (1995) and Cleeremans (2005), there are two functional aspects of consciousness, Access Consciousness, and Metacognition, when we, as argued above, leave Phenomenal Consciousness aside.

One function of Access Consciousness, as discussed here, is to allow the agent becoming independent of the hard-wired reactive structure by which memory elements can only be selected within a given context. This is, for instance, required if a behavioral problem occurs, i.e., a situation not treatable by the existing system. In the state of Access Consciousness, the agent is able to plan ahead, and thereby to test new ideas, i.e., new combinations of elements of the procedural memory. These new ideas, when successfully tested by internal simulation, are used to guide the newly invented behavior.

The advantages for an agent endowed with properties of Access Consciousness come with drawbacks: (i) Controlling behaviors through a conscious state is slower than controlling it by reactive structures. (ii) Application of consciousness allows for inventing new behaviors, but, when being activated during an ongoing reactively controlled action, might worsen the performance. Both properties can also be found in psychological experiments with human participants.

The architecture used here, that allows to control behavior and endorses properties of Access Consciousness, may also be suited to set the stage for the later introduction of neural structures that can function as neural representation of – averbal and verbal – concepts. However, here we concentrated on a specific domain, solving motor problems. Such problems cover an area being less restricted than it might seem to be the case at a first glance, as many problems, including abstract mathematical problems, can arguably be understood as being based on the ability to solve motor tasks (e.g., Lakoff and Núñez, 2000; Glenberg and Gallese, 2012). In addition to being concerned with motor control, reaCog might be confronted with situations that might be seen as to belong to perception and where attention may not be driven by the WTA system of the “attention controller.” For instance, an unexpected stimulus may, in a bottom-up fashion, direct attention to a memory element that represents this kind of stimulus. Similarly, top-down attention is possible. The latter would however require further structures to represent the above mentioned averbal or verbal concepts not yet introduced in reaCog.

Another aspect, not covered by the simple structure of reaCog, concerns incubation (Helie and Sun, 2010). Incubation might help when a problem is given for which actually no solution can be found. A sensible way out of such a deadlock might be to quit the current goal and introduce another one. As for the simple version of reaCog discussed here, internal simulation is only possible whilst the actual behavior is interrupted, switching the goal means that the problem as such would remain unsolved. Incubation describes the observation that humans, in contrast to reaCog, can apparently search for solutions even if other behaviors are active. Thus, a further challenge is to introduce structures that allow searching for solutions of open problems, whilst the agent is performing other behaviors.

Apart from such specific shortcomings that arise when trying to compare a simple system like reaCog with fully conscious systems as humans, a more general counterargument might be to consider Block's conceptualizations that we use here as a scaffold for helping to understand consciousness, as basically misguided. Following this view, properties of reaCog might still be considered interesting, but of minor relevance for the discussion of what is meant by consciousness. One specific case is represented by authors who, as reviewed by Lau and Rosenthal (2011) restrict consciousness to Metacognition only, and are not prepared to attribute properties of consciousness to what is termed Access Consciousness by other authors. This view represents a challenge to expand reaCog for endorsing properties of Metacognition.

Metacognition, or reflexive Cognition, addresses the ability to deal with own mental states. A related aspect has been described by the term Theory of Mind, which characterizes the ability to attribute mental states (e.g., emotional states) also to other agents (Premack and Woodruff, 1978). This has often be described as the ability to “step into the shoes of the other.” In classical experiments, this capability is tested in the so-called Sally–Anne task. Two subjects are shown that a candy lying on the table is hidden under a black cover. Then one subject, Sally, has to leave the room whilst the candy is now hidden under the white cover, as observed by Anne. After Sally has come back, Anne is asked under which cover Sally will probably search for the candy. If Anne points to the black cover, she is assumed to have a Theory of Mind, but not, if she points to the white cover where the candy really is placed. Being endowed with the faculty of applying a Theory of Mind would allow to better model the world when it contains not only mere physical objects but other agents capable of operating with not directly observable plans and intentions. Thus, the ability to attribute a Theory of Mind, or mental states, to others allows the agent to better predict the behavior of the other. Two main alternative explanations are discussed as to how Theory of Mind is realized. The so-called theory–theory (Carruthers, 1996) assumes that there are (innate) procedures that allow for prediction of others. In contrast, simulation theory (Goldman, 2005) assumes that the agent has an internal model of him or herself that can be used to represent the other, too. Via internal simulation (or “probehandeln”), this model can simulate the behavior of the other agent, based on the properties of the simulating agent. However, both theories are not necessarily excluding each other. If we assume that reaCog is expanded by a network that allows to use its own body model to represent another agent (see Cruse and Schilling, 2011) for a sketch of how such a network may be constructed), this model could be used for the simulation. If such a simulation has led to a new, successful interpretation of the behavior of the other, the result could be stored as a procedure, as described for reaCog when having learnt new solutions. In this way, the simulation result could be stored as part of the reactive memory complementing the already existing innate procedures. In this way, the structure allowing for internal simulation may provide a tool for enriching the procedures usable to predict the behavior of others. In any case, the faculty to apply a Theory of Mind is clearly beyond the ability of reaCog, which allows for an egocentric view only.

### Conflict of interest statement

The authors declare that the research was conducted in the absence of any commercial or financial relationships that could be construed as a potential conflict of interest.

## Footnotes

^1^In a recurrent winner-take-all network, each unit receives positive feedback from itself and negative feedback from all other units of the network. When any random activation is given to these units, after some iterations one unit will show a positive activation and all other units will show an activation of zero.

^2^A low-pass filter is characterized by an increase of output activation that, when excited by a constant stimulus, asymptotically approaches a given output value. Such low-pass filter dynamics are for example given by RNN with attractor properties. In this case, the so-called harmony value (Rumelhart and McClelland, 1986) of the network can be used to characterize its state.

^3^There are various views adopting a monist approach differing in detail (epiphenomenalism, emergentism, property dualism and their many derivatives, see Vision, 2011). We will not take part in this discussion here.

^4^Using a WTA network this way has been termed biased competition (see e.g. Bundesen et al., 2011.

^5^we have not yet implemented the learning procedure in reaCog.

^6^Here, the term cognition is used in a more general way compared to the strict definition proposed by McFarland and Bösser (1993) and used in this article.
